# Multi-Analytical Investigations of Andy Warhol’s “*Orange Car Crash*”: Polymeric Materials in Modern Paints

**DOI:** 10.3390/polym14030633

**Published:** 2022-02-07

**Authors:** Valentina Pintus, Anthony J. Baragona, Federica Cappa, Christa Haiml, Christina Hierl, Katja Sterflinger, Manfred Schreiner

**Affiliations:** 1 Institute of Science and Technology in Art, Academy of Fine Arts Vienna, Schillerplatz 3, A-1010 Vienna, Austria; f.cappa@akbild.ac.at (F.C.); k.sterflinger@akbild.ac.at (K.S.); m.schreiner@akbild.ac.at (M.S.); 2 Institute for Conservation-Restoration, Modern-Contemporary Art, Academy of Fine Arts Vienna, Schillerplatz 3, A-1010 Vienna, Austria; 3 Institute of Art and Technology, Department of Conservation Science, University of Applied Arts, Salzgries 14, A-1010 Vienna, Austria; tonybaragona@gmail.com; 4 Conservation Department, Museum Moderner Kunst Stiftung Ludwig Wien (Mumok), A-1010 Vienna, Austria; christa.haiml@gmail.com (C.H.); tina.hierl@mumok.at (C.H.)

**Keywords:** *Orange Car Crash*, Andy Warhol, Py–GC/MS, THM–GC/MS, µ-Raman, µ-ATR–FTIR, modern materials, silkscreen

## Abstract

This work presents strategic multi-analytical investigations performed on “*Orange Car Crash*” by Andy Warhol in order to make a well-informed conservation decision. For determining the type of binding medium used in the artwork, Pyrolysis–Gas Chromatography/Mass Spectrometry (Py–GC/MS) and Thermally Assisted Hydrolysis and Methylation of GC/MS (THM–GC/MS) were employed. The presence of a coating was investigated by Py–GC/MS. Moreover, the comprehension and elucidation of the paint stratigraphy were studied by examining cross-sections of samples taken from both canvases with Optical Microscopy (OM) under reflected visible (Vis) and ultraviolet light (UV) and by Scanning Electron Microscopy with Energy Dispersive X-ray spectroscopy (SEM–EDX). The investigation of possible synthetic organic pigments (SOPs) and extenders was performed by µ-Raman spectroscopy, while micro-Attenuated Total Reflection of Fourier-Transform Attenuated Total Reflection (µ-ATR–FTIR) allowed us to assign each component detected by Py–GC/MS or THM–GC/MS to a specific layer. The data collected from “*Orange Car Crash*” show mostly the application of acrylic-based paint as well as alkyd with rosin acids-based ink, thus providing fundamental information about the paint stratigraphy and chemical composition of each layer. In addition to the goal of informing an appropriate conservation–restoration strategy, this work represents a rare scientific study of a work by Andy Warhol.

## 1. Introduction

### 1.1. “ Orange Car Crash” by Andy Warhol in the Time of Modern Materials

**Figure 1:**
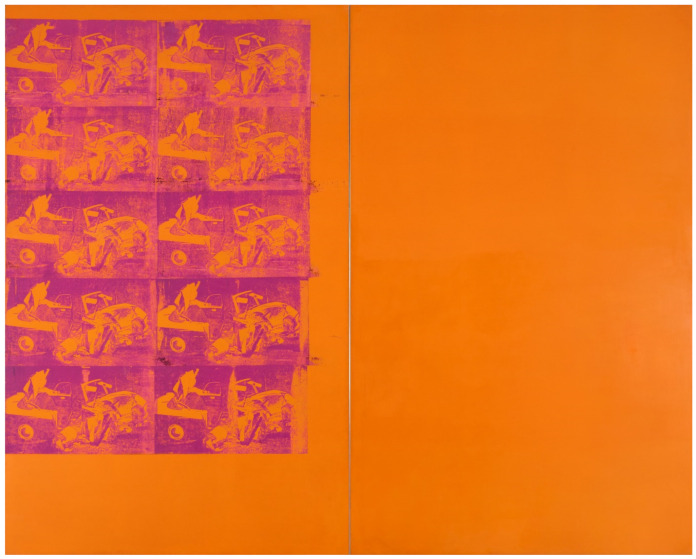
“*Orange Car Crash*” by Andy Warhol based on a silkscreen canvas (left) paired with a monochrome canvas (right). Photo © mumok—Museum Moderner Kunst Stiftung Ludwig Wien, on loan from the Ludwig collection, Aachen.

“*Orange Car Crash*” by Andy Warhol (1928–1987) consists of two canvases (334 × 412 cm), a silkscreen paired with a monochrome (Figure 1). It is a major work in the mumok (Museum Moderner Kunst Stiftung Ludwig Wien) in Vienna (Austria), on loan from the Ludwig collection Aachen (Germany).

There are a number of examples of Warhol pairing a silkscreen with a monochrome canvas (Warhol referred to such canvases as “blanks”); in most cases, the monochrome canvases were added at a later date, when the work was sold from the Warhol studio or when they were first exhibited. “*Orange Car Crash*” is dated 1963 and was equally not conceived as a diptych from the beginning; the monochrome canvas was added later on. The biography of the monochrome canvas has not yet been completely resolved, but it is likely that it was added in the late sixties, when it was first sold from the Warhol studio. This assumption is supported by the fact that the silkscreen and the monochrome are executed on different types of canvas (different canvas weights and different colored selvage), and that they vary in the build-up and composition of the ground layers. No difference could be discerned in the orange paint layer.

Warhol started to use acrylic paint in early 1962. Before then, he had mainly been using casein paint and other water-based paints (such as watercolor or ink), and possibly oil paint in some instances [1]. Acrylic paint had been available in America for a few years by then, but had only become more widely used by artists around 1963 [2]. In the form of waterborne emulsions, artist acrylic paint has been revolutionary in the paint and coating sector because of its quick-drying and environmentally friendly properties, and because of the presence of water instead of organic solvents. Based on an ester of acrylic and methacrylic acids, acrylics are commonly characterized by three main different co-emulsions: poly(Ethyl Acrylate/Methyl Methacrylate (EA/MMA), mainly found in the early years, poly(*n*Butyl Acrylate/Methyl Methacrylate) (*n*BA/MMA), introduced in the late 1980s, and, in recent years, 2-Ethylhexyl Acrylate/Methyl Methacrylate (2-EHA/MMA).

Warhol’s switching to acrylic paints in 1962 coincides with his first silkscreened paintings, the *Dollar Bills Series* [1]. These were outlined in ink on acetate sheets and hand cut, but Warhol quickly started incorporating black and white photographs using a photomechanical process [1,2]. In photo silk-screening, the source is transferred onto a silkscreen coated with a light-sensitive emulsion that becomes insoluble when exposed to light. The unexposed areas, however, can be re-dissolved and washed off. This is a process that was carried out for Warhol by a silk-screener. Warhol then used the prepared screen and applied a silkscreen medium through it using a squeegee. In the case of “*Orange Car Crash*”, Warhol used a commercially available silkscreen ink, indicated by the alkyd resin as a binder. Alkyd resin also represented an innovative material. It started to be developed and made commercially available in the early 1930s [3], and it is mostly based on a polyester conformation formed by a polyhydric alcohol and a polybasic carboxylic acid with the addition of monobasic fatty acids.

In earlier silkscreens, Warhol is also known to have used a silkscreen medium that he made himself in the studio, mixing acrylic paint and glycerin [1]. The ink could only be pushed through the screen in areas where the unexposed emulsion had been washed off, and was thus deposited as a pattern of dots. 

### 1.2. Challenges in Modern Materials Analyses

Acrylic and alkyd paints represent a large group of modern materials as a great part of artworks in many different museum collections around the world. In order to assess and develop conservation strategies for modern and contemporary art collections, the identification and characterization of their main materials through scientific analyses is a crucial step. The very high complexity of the chemical composition of modern and contemporary art paint colors, which includes mainly synthetic organic materials used as binders as well as additives normally present only in a very low concentration, makes their characterization by scientific investigation quite challenging. Over time, many different worldwide manufacturers have produced modern paint materials and have constantly improved and/or modified the chemical composition of their products in order to meet artists‘ requirements. Therefore, a precise identification of the paint material may also be of help for dating purposes. Additionally, the main chemical composition of modern art materials may undergo photo-oxidation and/or degradation processes, resulting in different chemical compounds that are difficult to identify. Another challenge is when a complex or multilayered paint stratigraphy with the addition of a coating is present in an artwork, in which the identification and characterization of each layer require sampling and, in general, the synergetic use of different analytical techniques.

Numerous scientific studies have been reported so far on the analyses of modern paints [4,5,6,7,8,9,10]. Fourier-Transform Infrared spectroscopy (FTIR) is a well-established and an optimal technique for defining the chemical class of the binders found in modern paint formulations, such as acrylic, alkyds, etc., while Pyrolysis–Gas Chromatography–Mass Spectrometry (Py–GC/MS) can provide deeper information about the chemical composition of the base polymer of the media. Scanning Electron Microscopy with Energy Dispersive X-ray spectroscopy (SEM/EDX) has been reported in numerous studies for the investigation of inorganic pigments and/or extenders [11,12,13]. The addition of extenders is not only principally related to reducing the costs and increasing the volume of the material—extenders can also have beneficial effects on the paint by modifying the quality depending on their grain size, refractive index, brightness, and chemical composition. Different types of inorganic extenders, for example, talc (Mg_3_(Si_4_O_10_)(OH)_2_), barite (BaSO_4_), calcite (CaCO_3_), kaolinite (Al_2_Si_2_H_4_O_9_), and various forms of silica, are commonly used. The demand for various types of extenders for use in paints is increasing because of their ability to modify the quality of paints depending on their grain size, refractive index, brightness, and chemical composition. Additionally, Raman spectroscopy has been extensively used for the identification of colorants and pigments [14,15]. 

So far, little information is available about the exact chemical composition of Andy Warhol’s paint materials, rather than citing that acrylics and inks were used. The use of a p(EA/MMA) as acrylic paint was identified in four canvasses of Andy Warhol’s *Portrait of Brooke Hayward* (1973) together with different types of synthetic organic and inorganic pigments via Fourier-Transform Infrared spectroscopy (FTIR), Energy Dispersive X-ray spectroscopy (EDX), and Pyrolysis–Gas Chromatography–Mass Spectrometry (Py–GC/MS) [16]. On the other hand, the priming and the silkscreen ink were identified as oil-modified alkyd media [16]. Another study reported that solvent-sensitive inks were found on several hand-colored lithographs from an edition of Andy Warhol’s *A la Researche du Shoe Perdu* portfolio, c. 1955 [17]. Those inks were characterized by FTIR and High-Pressure Liquid Chromatography (HPLC) as mixtures of various synthetic dyes [17].

### 1.3. Aim of This Research

In this work, an in-depth multi-analytical investigation was carried out on samples acquired from the two canvases of “*Orange Car Crash*” by Andy Warhol, with the aim of informing conservation–restoration strategies. A major focus of the examination of the monochrome canvas was on a non-original coating that has been selectively applied to it. For this purpose, samples were selected from both canvases to establish and compare their stratigraphy. Both canvases were first examined with visible and UV light and by a microscope. The non-original coating was visible in UV light, but was also readily apparent in normal light because of its milky appearance and uneven application, which is visually disturbing in certain light situations. Samples of the paint and ground layers were taken from areas on the tacking edges that were already detaching, a sample of the coating was taken by scraping, and a sample of the silkscreen paint was taken from another detachment. For this study, Pyrolysis–Gas Chromatography/Mass Spectrometry (Py–GC/MS) and/or Thermally assisted Hydrolysis and Methylation of GC/MS (THM–GC/MS) were employed for the identification and characterization of the type of binding medium of each canvas and possible presence of a coating. Additionally, optical microscopy (OM) in visible (Vis) and ultraviolet (UV) light, Scanning Electron Microscopy–Energy Dispersive X-ray (SEM–EDX) spectroscopy, and µ-Raman spectroscopy were used for investigating the paint stratigraphy and inorganic components included, such as fillers and pigments, and the synthetic organic pigments (SOPs) by µ-Raman spectroscopy. Furthermore, µ-Attenuated Total Reflection of Fourier-Transform Infrared (μ-ATR–FTIR) spectroscopic analyses were conducted for the investigation of both the organic and inorganic main components in the paint stratigraphy. 

## 2. Experimental

### 2.1. Materials 

Several samples were taken from the two canvases of “*Orange Car Crash*”. The description and labeling of the received and analyzed samples are reported in Table 1.

### 2.2. Optical Microscopy (OM) and Scanning Electron Microscopy with Energy Dispersive X-ray Spectroscopy (SEM/EDX) 

The stratigraphy of two cross-sectioned samples of the monochrome canvas (OM P5 and OM P6) and three cross-sectioned samples of the silkscreen canvas (OS P3, OS P4, and OS P5) was studied with both OM and SEM/EDX. To prepare cross-sections, a tiny and representative fragment was taken from the selected samples and embedded into epoxy resin (type Araldite 2020/A) with an amount of 3% of hardener (type 2020/B, Huntsman, USA). In order to observe the stratigraphy under OM without any interference of a rough surface, the cross-sections were polished by using abrasive grinding paper based on silicon carbide (SiC) with different grit sizes (from 600 to 12,000 grits) after drying/hardening for 5 days at room temperature. 

For the optical microscopy, a ZEISS Axioplan 2 Imaging microscope (Oberkochen, Germany) (10×, 20×, and 50× objectives and 10× oculars) was employed by using visible (Vis) as well as UV radiation (UV lamp HBO 100, OSRAM, Munich, Germany). Images of the samples were acquired by a Nikon D700 Camera and then collected and evaluated with the Camera Control Pro2 Software. SEM/EDX analysis was performed using a Quanta FEG 250 (FEI, Hillsboro, OR, USA) scanning electron microscope coupled to an Octane Elect Plus EDX detector (Ametek/EDAX, Berwyn, IL, USA) equipped with the Genesis EDX Quant software. The investigations were performed under a low vacuum at an accelerating voltage of 20 kV in the back-scattered electron (BSE) detection mode. 

### 2.3. Pyrolysis–Gas Chromatography/Mass Spectrometry (Py–GC/MS) and Thermally Assisted Hydrolysis and Methylation–Gas Chromatography/Mass Spectrometry (THM–GC/MS)

Pyrolysis–Gas Chromatography/Mass Spectrometry (Py–GC/MS) analysis was performed on samples OM P1, OM P2, OM P4, OS P1, OS P2, and OS P4 (Table 1). Additionally, THM–GC/MS (Thermally assisted Hydrolysis and Methylation) analytical mode based on Py–GC/MS analysis was selected for the OM P3, OS P2, and OS P4 samples (Table 1). The latter method was chosen to better detect the possible presence of certain polar compounds, particularly in drying oils, such as large acids, by lowering their polarity with the use of the tetramethylammonium hydroxide (TMAH) methylating reagent. TMAH reagent acts when in contact with the sample and during the pyrolysis step, converting both ester-linked and free fatty acids to their respective methyl esters according to the reaction. For the THM–GC/MS analysis the sample material (around 0.22 mg) was put into a sample cup (ECO-CUP Frontier Lab, Korijama, Japan) and treated with 2 μL of tetramethylammonium hydroxide (TMAH) reagent (25 wt% aqueous solution of TMAH, Sigma Aldrich, Milwaukee, WI, USA.).

For the analyses of the samples, a pyrolyzer PY-2020iD (Frontier Lab., Korijama, Japan) combined with a GCMS-QP2010 Plus (Shimadzu, Kyoto, Japan) was employed. The GC/MS unit was equipped with a capillary column SLB-5ms Supelco, U.S.A. (30 m length × 0.25 mm internal diameter × 0.25 μm film thickness) using bonded and highly cross-linked 5% diphenyl/95% dimethyl siloxane. The capillary column was connected to a deactivated silica pre-column Rxi Guard Column Restek, USA (5 m length × 0.32 mm internal diameter). The NIST 05 and NIST 05s Library of Mass Spectra were available for the identification of the compounds. 

For both the Py–GC/MS and THM–GC/MS analyses, the pyrolysis temperature was set at 600 °C, while the pyrolysis interface and the injector temperature were set at 280 °C and 250 °C, respectively. The GC column temperature conditions used for both Py–GC/MS and THM–GC/MS were as follows: initial temperature 40 °C, held for 2 min followed by a temperature increase of 6 °C/min to 300 °C for 20 min. The helium gas flow was set at 1 mL/min and the electronic pressure control was set to a constant flow of 31.7 mL/min in the split mode at a 1:50 ratio. The mass spectra were recorded under electron impact (EI) ionization in the positive mode at 70 eV and the temperatures of the MS interface and the ion source were 280 °C and 200 °C, respectively. The mass spectrometer scanned from 50 m/z to 750 m/z. For the THM–GC/MS, a solvent cut time of 5 min by turning off the filament in the ion source was used. This mode prevents the sharp decrease of the vacuum inside the ion source due to the entrance of the TMAH reagent, which has a detrimental effect on the filament and other components.

### 2.4. Micro-Attenuated Total Reflection of Fourier-Transform Infrared (µ-ATR–FTIR) Spectroscopy

µ-ATR–FTIR analyses were performed with a LUMOS standalone FTIR microscope (Bruker Optics GmbH, Bremen, Germany) equipped with a Globar thermal light source, a RockSolidTm interferometer, and a liquid nitrogen-cooled mid-band 100 × 100 µm^2^ Photoconductive Mercury–Cadmium Telluride (PC–MCT) detector. The ATR probe was a germanium frustum cone-shaped crystal (Ge, refractive index *n* = 4) with a tip diameter of 100 µm. This ATR probe was implemented into a fully motorized and automated piezo motors 8× Cassegrain objective (NA = 0.6). An XYZ motorized sample stage allows selecting a priori the applied pressure of the ATR probe during the measurements in three different modes, such as low, medium, and high. Due to the sensitivity of the cross-section material, a low pressure was selected, which did not leave any microscopically recognizable print on the surface of the sample. All the optics and beamsplitter were made of zinc selenide (ZnSe). Spectra were acquired in the spectral range between 4000 and 600 cm^−1^, performing 128 scans at 4 cm^−1^ resolution. The resulting spectra were collected and evaluated with the spectrum software OPUS-IR^TM^ (Bruker Optics GmbH, Bremen, Germany, Version 8.1).

### 2.5. µ-Raman Spectroscopy

µ-Raman measurements were performed in a dark room using a ProRaman-L-Dual-G analyzer (Enwave Optronics, Inc., Irwine, USA). This instrument is a fully integrated and portable Raman spectrometer. The excitation source applied in this study was a diode laser at 785 nm (~350 mW) with a narrow line-width of 2.0 cm^−1^. The fiber optic probe had a standard working distance of 7.5 mm, and it was correlated with a Rayleigh filter. The detector was a two-dimensional CCD array temperature regulated (−60 °C) detector. The spectral coverage ranged from ~100 cm^−1^ to 3300 cm^−1^. The integrated microscope was equipped with a 1.3 Mpixel camera with in-line LED illumination. The various areas of interest were measured using a Leica 50× (LWD) microscope objective (spot size ~3 µm). The spectra were collected and evaluated with the spectrum software OPUS (Bruker Optics GmbH, Bremen, Germany, Version 7). The evaluation was performed by comparing the measured spectra with those of a database (Institute of Natural Sciences and Technology in the Art (ISTA) reference database).

## 3. Results and Discussion

### 3.1. Monochrome Canvas

**Figure 2:**
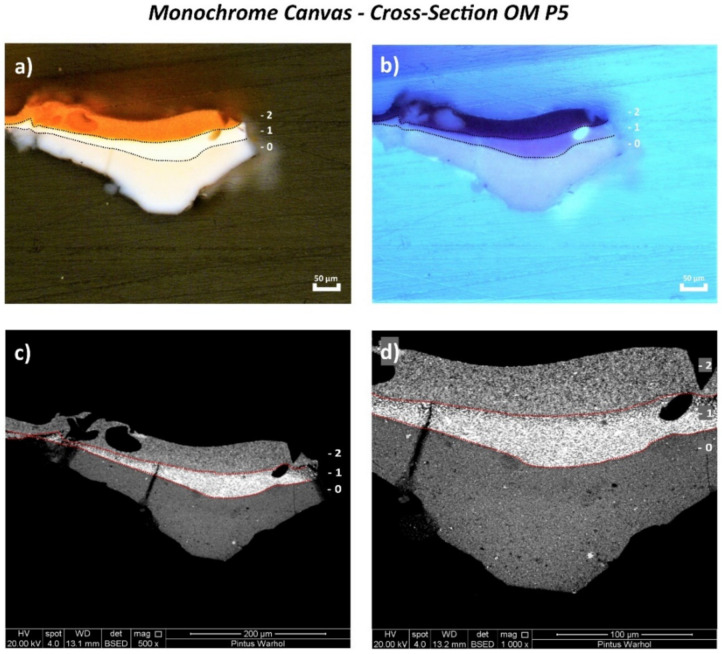
(**a**) Optical microscopy image of the cross-sectioned OM P5 sample under reflected visible light (Vis) showing the main layers and (**b**) under ultraviolet light (UV). (**c**) SEM micrograph of the whole cross-sectioned OM P5 sample and (**d**) detail of the area investigated by bulk-area EDX analyses showing the main paint layers.

**Figure 3:**
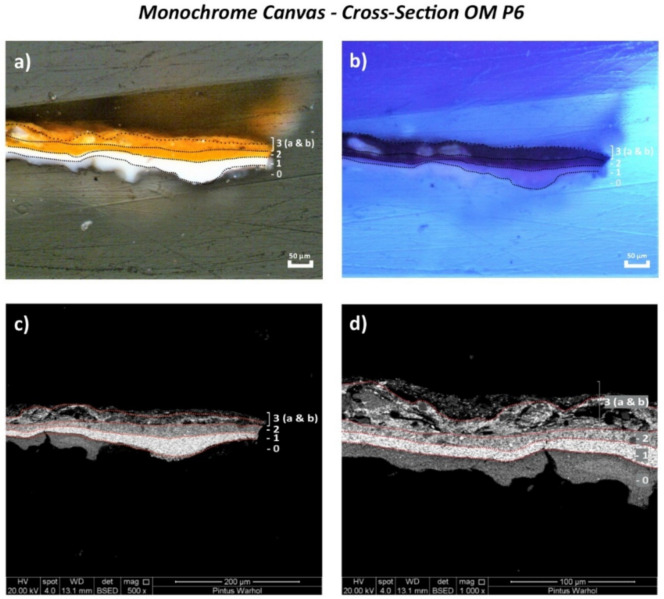
(**a**) Optical microscopy image of the cross-sectioned OM P6 sample under reflected visible light (Vis) showing the main layers and (**b**) under ultraviolet light (UV). (**c**) SEM micrograph of the whole cross-sectioned OM P6 sample and (**d**) detail of the area investigated by bulk-area EDX analyses showing the main paint layers.

Examination of the cross-sectioned stratigraphic samples (OM P5 and OM P6) of the monochrome “*Orange Car Crash*” canvas by the Optical Microscope (OM) using reflected visible light (Figure 2a and Figure 3a, respectively) showed the application of two different white ground layers (0 and 1st layers) with an orange homogeneous layer (2nd layer) on the top (OM P5). An additional orange layer (3rd layer)—ascribable to a retouching—was found in OM P6. This is shown in Figure 3, which appears characterized by two layers (a and b). These two layers are shown by the Scanning Electron Microscopy (SEM) micrographs (Figure 3c,d) with different consistencies based on the less dense appearance of layer 3b in comparison to that of layer 3a.

The two white ground layers of OM P5 and OM P6 were better distinguished by ultraviolet light (UV) under the OM (Figure 2b and Figure 3b), whereas the different layers of the complete paint stratigraphy were visualized best by the SEM micrographs (Figure 2c,d and Figure 3c,d). The main chemical components identified for each layer are summarized in Table 2 and fully described hereafter.

GROUND WHITE LAYERS: While the *first ground layer* (0 layer) was characterized by a mixture of *zinc white (ZnO—Color Index PW4)* as the pigment and *linseed oil* as the binder, the *upper ground layer* (1st layer) was composed of *titanium white (TiO_2_ rutile—Color Index PW6)* and *lithopone (barite (BaSO_4_) + zinc sulfide (ZnS)—Color Index PW5)* as the pigments with *linseed oil* as the binder as well. The inorganic components such as the white pigments were identified along the stratigraphy of OM P5 and OM P6 by EDX bulk area analysis (Table 3) and further supported by the µ-Raman measurements.

**Figure 4:**
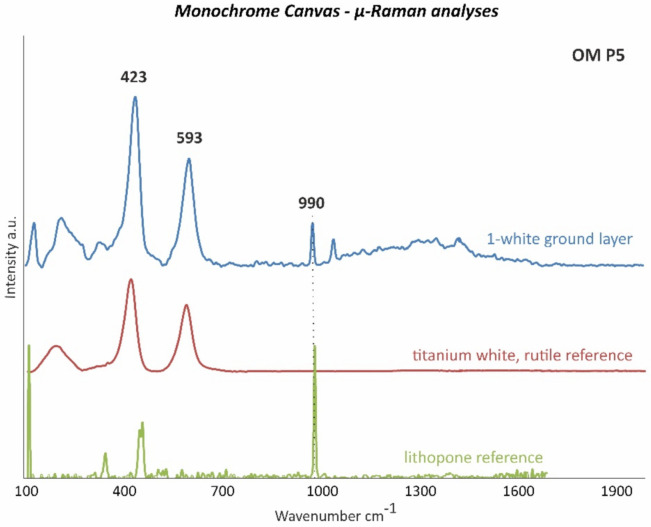
µ-Raman spectra of the white ground layer 1 of OM P5 (blue line) and of the titanium white, rutile (TiO_2_) (red line), and lithopone (BaSO_4_ + ZnO) (green line) references as the main detected components.

The differentiation between titanium white in either the rutile or anatase form in white ground layer 1 was accomplished by µ-Raman measurements. Thus, the more stable rutile form of titanium white was detected according to the bands at 423 and 593 cm^−1^ (Figure 4). Titanium white in the rutile form was firstly successfully synthesized in 1937, becoming increasingly available on the market during the second half of the twentieth century [18].

**Figure 5:**
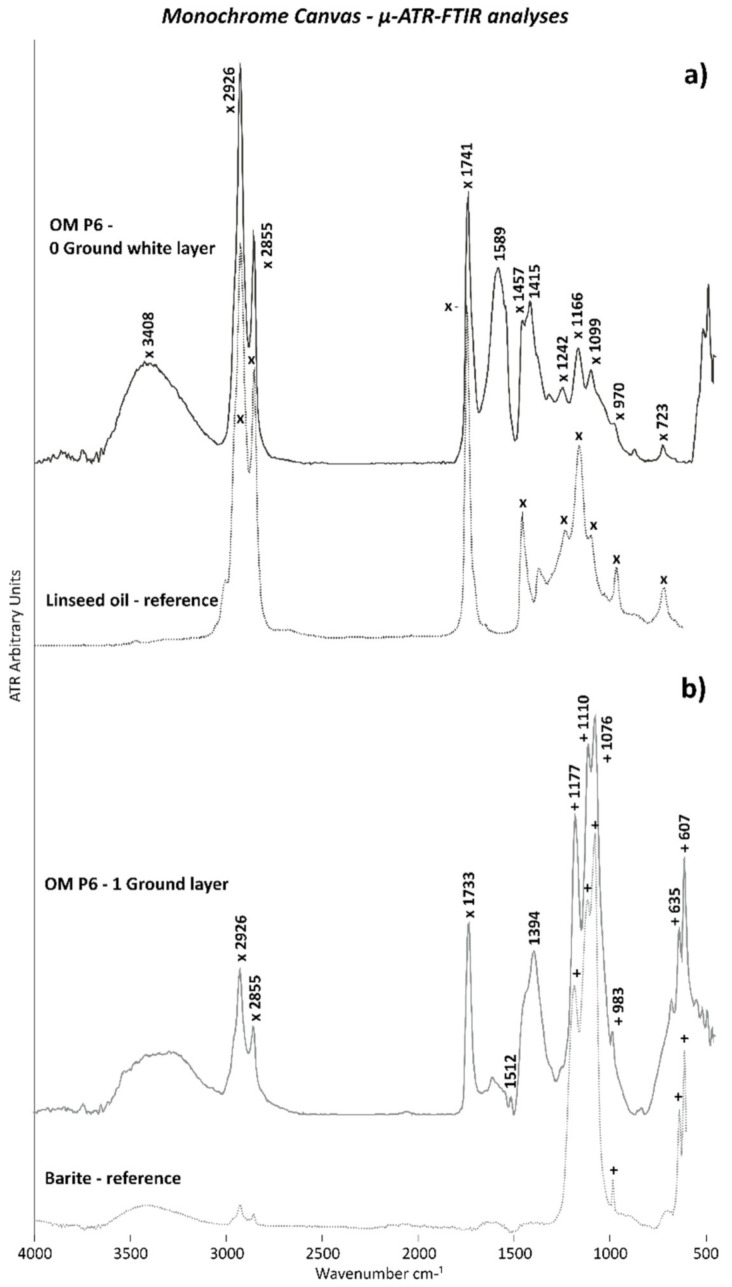
ATR spectra of white ground layers (**a**) 0 and (**b**) 1 of OM P6. The ATR spectra of the linseed oil (x) and barite (BaSO_4_) (+) references are also included.

The ATR spectra of the OM P5 and OM P6 samples indicated the presence of barite in the upper ground layer, as evidenced by its typical bands at 1173, 1110, 1076, and 983 cm^−1^ due to the S–O stretching mode and by the doublet at 633 and 605 cm^−1^ of the S–O bending mode (Figure 5b). 

**Figure 6:**
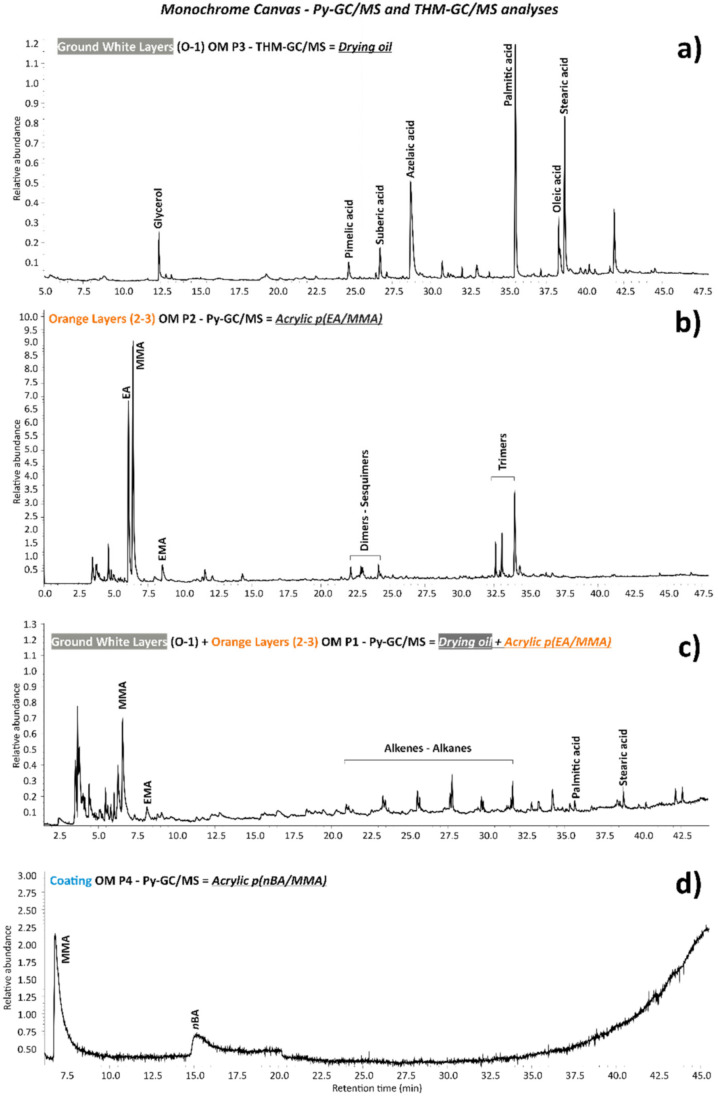
Pyrograms of the white ground layers (**a**) 0 and 1 of OM P3 sample acquired by TMH–GC/MS showing the most important marker peaks of a drying oil binder, (**b**) orange layers (2–3) of OM P2 sample by Py–GC/MS with the peaks of an EA/MMA acrylic binder, (**c**) both ground layers and orange layers of the OM P1 sample by Py–GC/MS with their respective peaks, and (**d**) coating/varnish layer of the OM P4 sample by Py–GC/MS depicting the main peaks of an nBA/MMA acrylic.

On the other hand, the chemical composition of the drying oil binder as an organic component was suggested by the Py–GC/MS analyses of the OM P1 sample containing both white ground layers and orange layers. This result was shown by the presence of saturated fatty acids, such as palmitic and stearic acids, and a series of alkenes/alkanes (Figure 6c), which are typically formed under the normal pyrolysis conditions of a drying oil binder. 

For confirming this hypothesis, THM–GC/MS analysis was performed on OM P3 containing only the two white ground layers. This analysis proved that a drying oil binder was part of the white ground layers, based on the characterization and identification of saturated acids, such as palmitic (*m*/*z* = 55, **74**, 87, 143, 171, 227, 270) and stearic acids (*m*/*z* = 55, **74**, 87, 143, 199, 255, 298), polyunsaturated acids, such as oleic acid (*m*/*z* = **55**, 69, 83, 98, 123, 137, 152, 166, 180, 222, 264), and diacids, such as pimelic (*m*/*z* = 55, 74, 83, 97, **115**, 128, 139, 157), suberic (*m*/*z* = 5, 69, **74**, 83, 97, 111, 129, 138, 171), and azelaic acids (*m*/*z* = **55**, 74, 87, 97, 111, 124, 143, 152, 185), which are shown in Figure 6a. Additionally, glycerol (*m*/*z* = **59**, 89, 102) from the triglyceride structure of the oil binder was registered. 

Generally, the relative amount of azelaic acid in comparison to palmitic acid (A/P) allows for the distinction between a drying oil and the egg lipidic fraction to be made, while the ratio of the palmitic and stearic (P/S) acids is an indicator of oils of a botanical origin (e.g., linseed, walnut, safflower, or poppy seed oil) [19,20,21,22,23,24]. However, both ratios are influenced by different factors, such as the instrument set-up used for the investigations, the type of pigment in which the binder is mixed, as well as the environmental conditions in which the artworks were kept over time. All of those factors could lead to an incorrect evaluation of the result. 

The A/P and P/S ratios of the investigated OM P3 sample corresponded to 0.75 and 1.45, respectively. The comparison of these values with those obtained in the literature [21,25,26,27,28,29] suggests the presence of linseed oil.

In order to confirm the presence of the linseed oil binder in both white ground layers, µ-ATR–FTIR investigations of the cross-sectioned samples OM P5 and OM P6 were performed. As shown in Figure 5a, a good match between the ATR spectra acquired on the white ground layer 0 of the cross-sectioned sample OM P6 with the linseed oil reference was clearly obtained. Indeed, the main IR bands of a linseed oil binder at 3408 (O–H stretching), 2926 and 2855 cm^−1^ (C–H stretching), 1741 cm^−1^ (C=O stretching), 1457 and 1415 cm^−1^ (C–H bending), 1242, 1166, and 1099 cm^−1^ (C–O–C stretching), 970 cm^−1^ (trans C=CH bending), and 723 cm^−1^ (cis C=CH bending) were detected. Furthermore, Figure 5a depicts the relatively lower intensity of the carbonyl band at 1741 cm^−1^ in comparison to the C–H sharp and intense peaks at 2926 and 2855 cm^−1^, which were found in the literature in the case of an oil binder mixed with zinc white pigment [30]. These results are thus in agreement with the organic and inorganic material composition of the 0 white ground layer.

The relatively high intensity and the sharp shape of the main IR bands at 1733, 2926, and 2855 cm^−1^ in the ATR spectrum acquired for white ground layer 1 also recall the presence of a linseed oil type of binder (Figure 5b). Particularly, the intense and broad band at 1394 cm^−1^ can be attributed to metal soaps, which are complexes of metal ions (e.g., Zn and Pb) and fatty acids generally formed by a reaction between the oil binder and inorganic pigments. 

Metal soap formation is also evidenced in white ground layer 0 of OM P6 (Figure 5a) by the broad and intense band at around 1589 cm^−1^, but also by the large band with a maximum at 1415 cm^−1^, both of which can be attributed to zinc soaps. 

ORANGE LAYERS: The *orange layer (2nd layer)* placed on top of the white ground layer 1 in the OM P5 and OM P6 is based on *cadmium sulfoselenide lithopone orange (CdS,xCdSe + BaSO_4_—Color Index PO20:1)* as the pigment and *EA/MMA acrylic* as the binder (copolymer of Ethyl Methacrylate and Methyl Methacrylate). On the other hand, the *additional orange layer (3rd layer)* found in OM P6—likely related to a retouching—is characterized also by *cadmium sulfoselenide lithopone orange (CdS,xCdSe + BaSO_4_—Color Index PO20:1)* as the pigment, *chalk (CaCO_3_)* as the extender, and *EA/MMA acrylic* as the binder. The inorganic components, such as the orange pigments and white extender, were identified along the stratigraphy of OM P5 and OM P6 by EDX bulk area analysis (Table 3).

Cadmium sulfoselenide lithopone orange belongs to the cadmium sulfide lithopone group of yellow and orange pigments first introduced by Marston in 1921 and obtained by co-precipitation f atrom solutions containing cadmium sulfate and barium sulfide [31]. Dark orange and red lithopones can be prepared by including selenium in the precipitation solution [32]. A cadmium yellow/orange with associated barium sulfate has been reported as Warhol´s palette in one of his later silkscreen artworks, such as “*Portrait of Brooke Hayward*” painted in 1973 [33,34]. 

**Figure 7:**
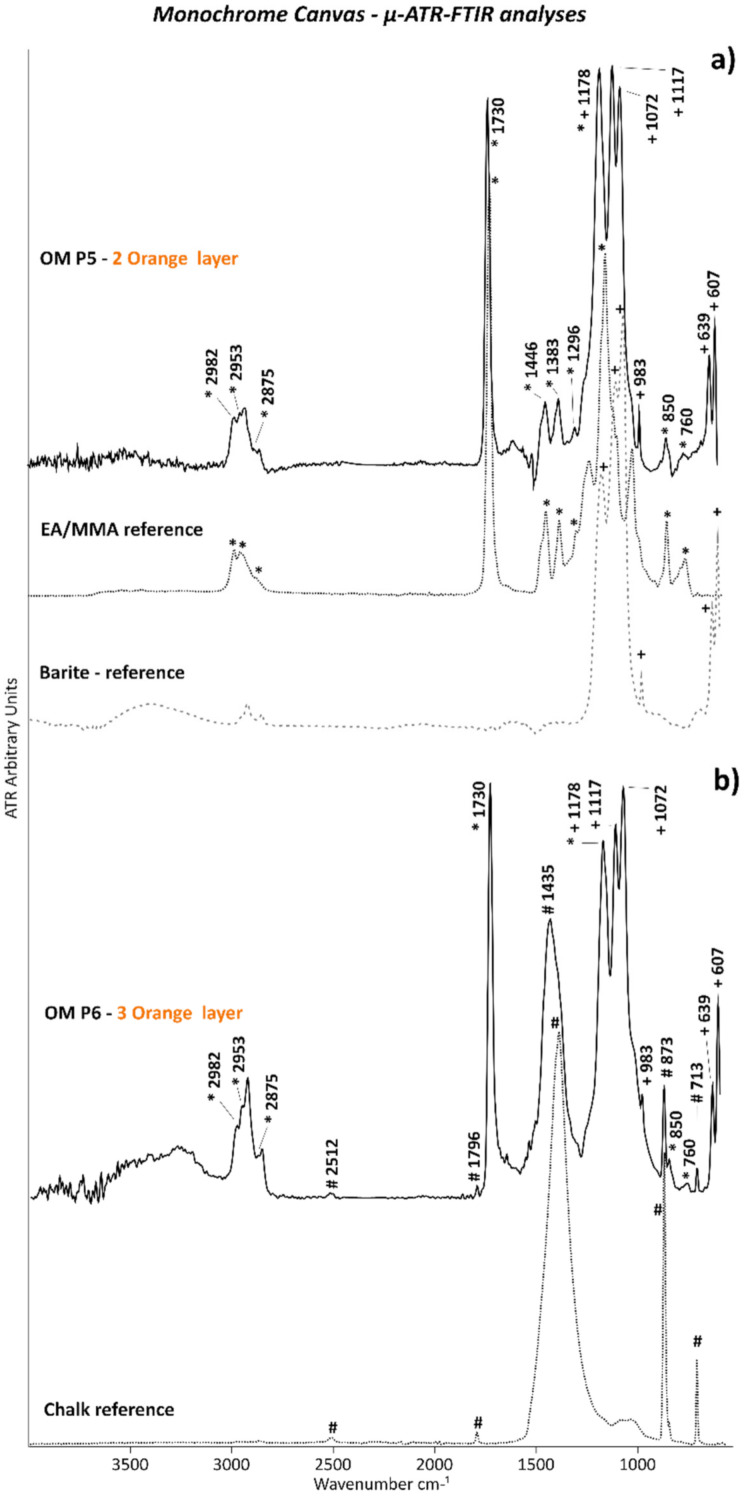
ATR spectra of orange layers (**a**) 2 and (**b**) 3 of OM P5 and OM P6, respectively. The ATR spectra of the EA/MMA acrylic (*), barite (BaSO_4_) (+), and chalk (CaCO_3_) (#) references are also included.

The presence of barite in both orange layers and chalk in the upper orange film were also proved by the µ-ATR–FTIR analyses based on their main typical IR bands (Figure 7a,b, respectively) (2512, 1796, and 1400 cm^−1^ because of C–O stretching; 873 and 762 cm^−1^ due to C–O bending of chalk; 1173, 1110, 1076, and 983 cm^−1^ of S–O stretching; and 633 and 605 cm^−1^ of S–O bending corresponding to barite). 

Regarding the organic component, EA/MMA acrylic binder was detected by Py–GC/MS in OM P2 containing the two orange layers. The main peaks of such an acrylic binder are shown in Figure 6b, which correspond to EA (*m*/*z* = **55**, 73, 85, 99) and MMA (*m*/*z* = 55, 59, **69**, 100) monomers, dimers (EA-MMA with *m/z* = **67**, 95, 140, 168, and EA with *m*/*z* = 53, **98**, 126, 154, 200), sesquimers (EA-MMA with *m*/*z* = **55**, 101, 128, 157, and EA with *m*/*z* = 55, 87, **143**, 162), and trimers (EA-EA-MMA with *m*/*z* = 55, **93**, 139, 166, 200, 255, 269 and 55, **93**, 121, 167, 200, 255, 269, and EA with *m*/*z* = 79, 106, **134**, 181, 208, 255) oligomers as typical pyrolysis products. Additionally, µ-ATR–FTIR analyses along the stratigraphy of the cross-sectioned OM P6 samples permitted to associate the EA/MMA acrylic binder to each of the two orange layers. Thus, the main IR bands of the EA/MMA acrylic binder at 2982, 2953, and 2875 cm^−1^ (C–H stretching), 1730 cm^−1^ (C=O stretching), 1446 and 1383 cm^−1^ (C–H bending), 1296 and 1178 cm^−1^ (C–O stretching), and 850 and 760 cm^−1^ (C–H rocking) were detected (Figure 7a,b). 

COATING: Sample OM P3, which was composed of tiny and few particles taken from the superficial part of the monochrome canvas, was investigated by Py–GC/MS and identified as an *n*BA/MMA acrylic (copolymer of *n*Butyl Methacrylate and Methyl Methacrylate). The main pyrolysis peaks of the *n*BA (*m*/*z* = **55**, 73, 85, and 128) and MMA (*m/z* = **69** and 100) monomers are highlighted in Figure 6d.

### 3.2. Silkscreen Canvas 

**Figure 8:**
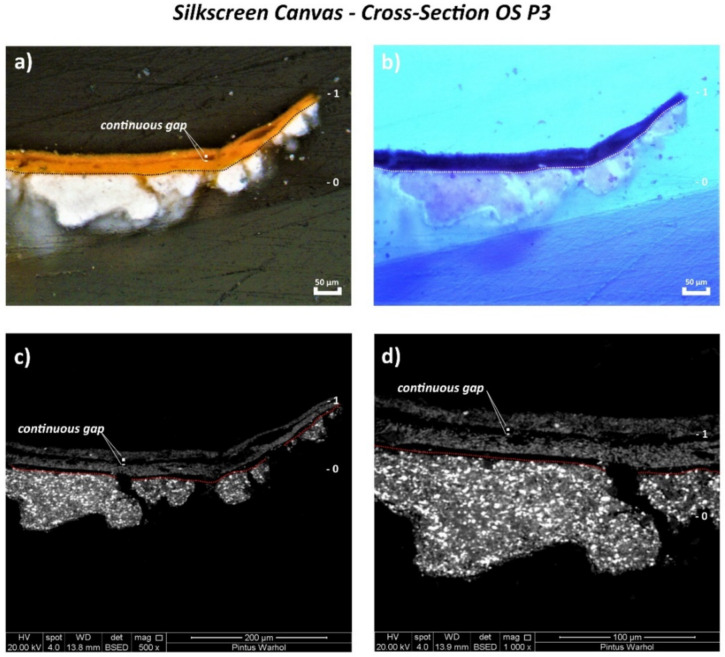
(**a**) Optical microscopy image of the cross-sectioned OS P3 sample under reflected visible light (Vis) showing the main layers and (**b**) under ultraviolet light (UV). (**c**) SEM micrograph of the whole cross-sectioned OS P3 sample and (**d**) detail of the area investigated by bulk area EDX analyses showing the main paint layers.

**Figure 9:**
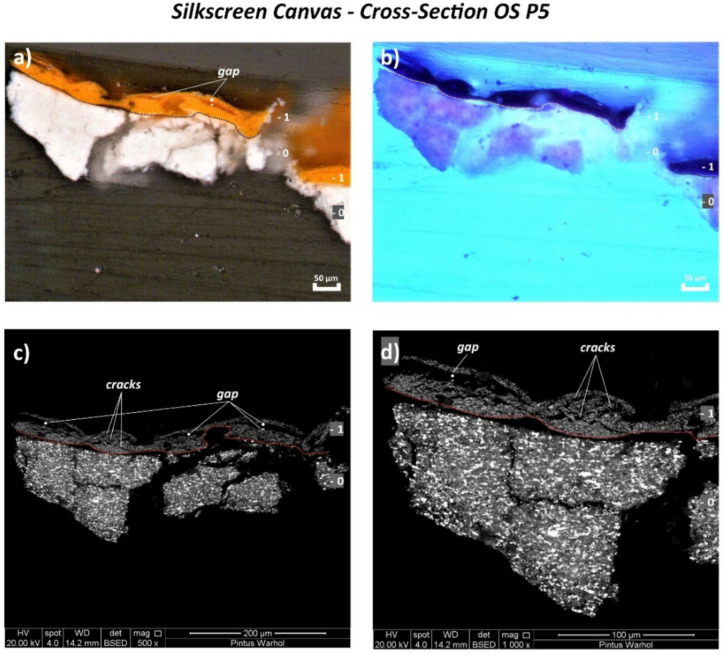
(**a**) Optical microscopy image of the cross-sectioned OS P5 sample under reflected visible light (Vis) showing the main layers and (**b**) under ultraviolet light (UV). (**c**) SEM micrograph of the whole cross-sectioned OS P5 sample and (**d**) detail of the area investigated by bulk area EDX analyses showing the main paint layers.

**Figure 10:**
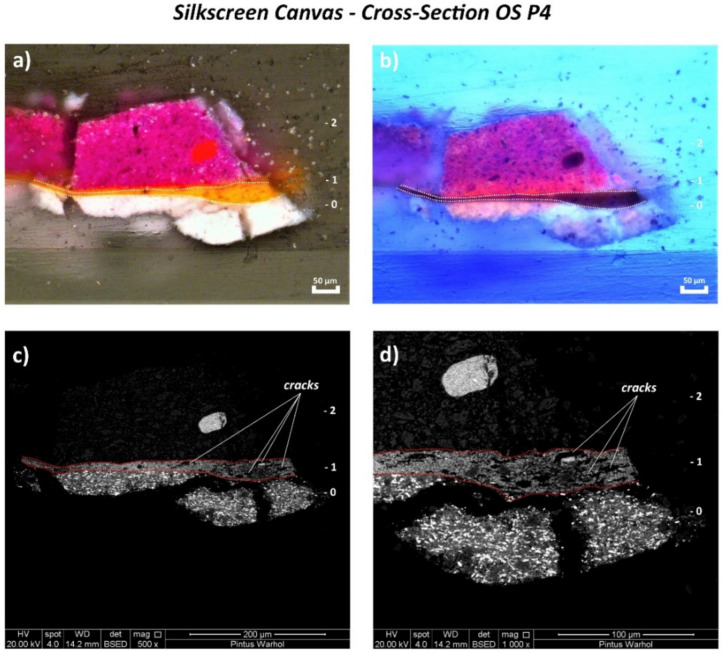
(**a**) Optical microscopy image of the cross-sectioned OS P4 sample under reflected visible light (Vis) showing the main layers and (**b**) under ultraviolet light (UV). (**c**) SEM micrograph of the whole cross-sectioned OS P4 sample and (**d**) detail of the area investigated by bulk area EDX analyses showing the main paint layers.

Contrary to the monochrome canvas, examination of the cross-sectioned stratigraphic OS P3, OS P4, and OS P5 samples of the silkscreen canvas by the Optical Microscope (OM) using reflected visible light (Vis) (Figure 8a, Figure 9a and Figure 10a, respectively) shows the application of only one white ground layer (0) instead of two. 

Only one orange layer (1st layer) was found on top of the white ground layer of all three cross-sections, while a further paint layer of violet color (2nd layer) was observed in OS P4 cross-section (Figure 10a). 

The orange layer of OS P3 and OS P5 appeared as two layers detached from each other with a continuous gap in between (Figure 8a and Figure 9a), likely as a result of the loss in adhesion of the paint layer. This physical effect is better depicted by the SEM micrographs in Figure 8c,d and Figure 9c,d. Moreover, the formation of several parallel cracks distributed from the top to the orange paint substrate was also visualized by SEM (Figure 9c,d and Figure 10c,d). 

The main chemical components identified for each layer are summarized in Table 4 and fully described hereafter.

GROUND WHITE LAYER: The white ground layer was characterized as being made of *titanium white (TiO_2_ rutile—Color Index PW6)* and *calcium sulfate (CaSO_4_—Color Index PW25)* as an extender mixed with a *soya oil* binder. 

The inorganic components, such as titanium white and calcium sulfate, were firstly detected by EDX element analyses (Table 5) according to their main elements. 

**Figure 11:**
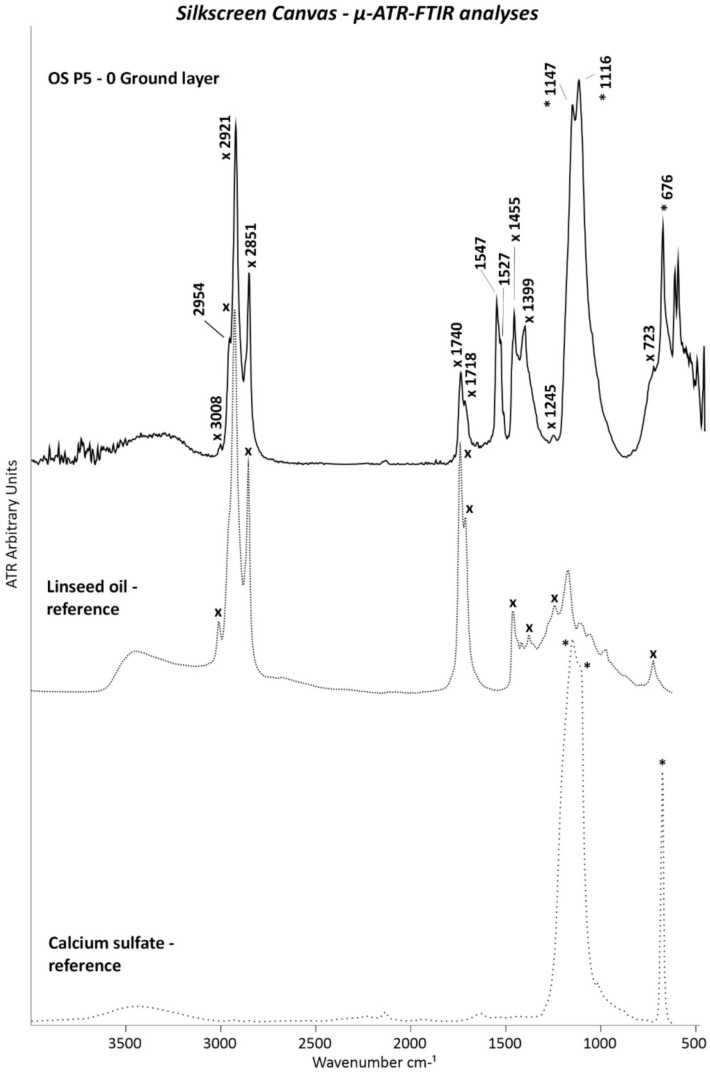
ATR spectra of white ground layer 0 of OS P5. The ATR spectra of the linseed oil (x) and calcium sulfate (CaSO_4_) (*) references are also included.

The presence of calcium sulfate was further supported by µ-ATR–FTIR analyses with the detection of its main IR bands at 1147, 1116, and 676 cm^−1^ (Figure 11). 

**Figure 12:**
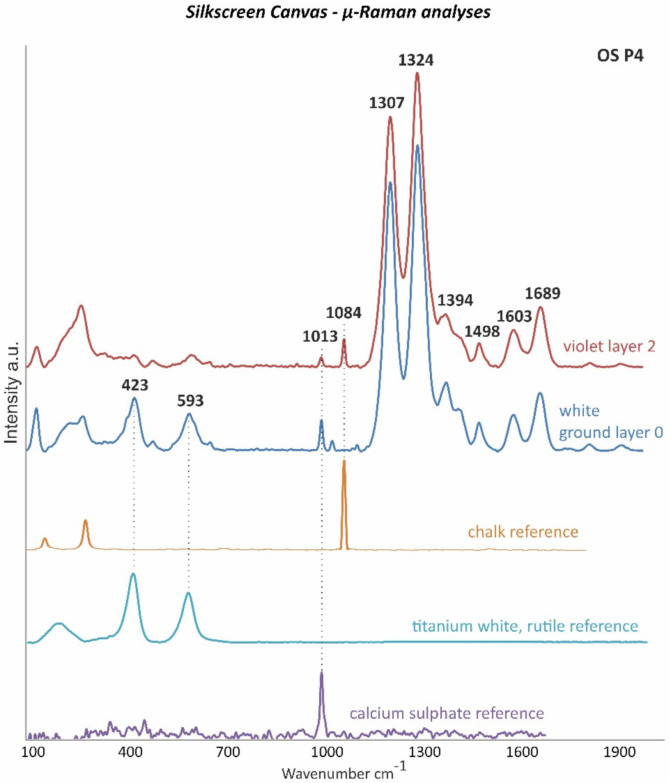
µ-Raman spectra of the white ground layer 0 (blue line) and the violet layer 2 (red line) of OS P4 and of the calcium sulfate (CaSO_4_) (violet line), titanium white, rutile (TiO_2_) (light blue line), and chalk (CaCO_3_) (orange line) references as the main detected components. Peaks at 1307, 1324, 1394, 1498, 1603, and 1689 cm^−1^ were not identified.

In addition to confirming the presence of gypsum based on the band at 1013 cm^−1^, µ-Raman measurements allowed for the characterization of the titanium white pigment as the rutile form based on its main bands at 423 and 593 cm^−1^ (Figure 12). 

**Figure 13:**
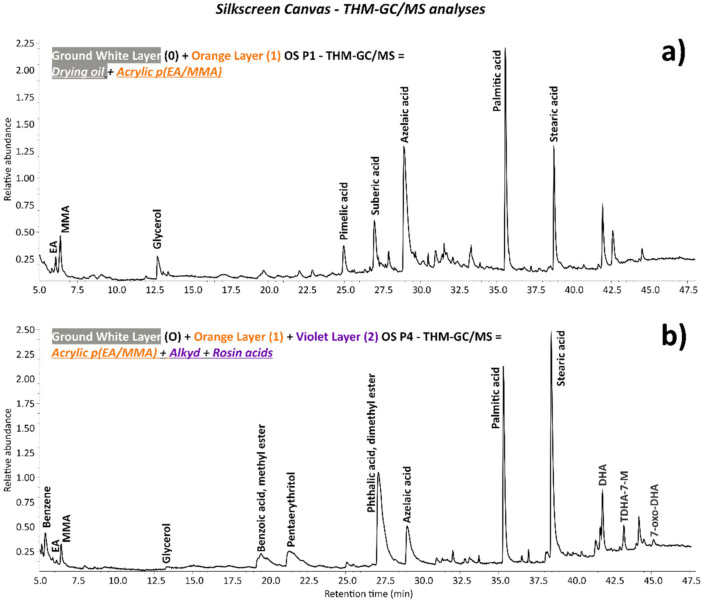
(**a**) Pyrogram of the white 0 and orange 1 layers of the OS P1 sample acquired by TMH–GC/MS showing the most important marker peaks of a drying oil binder (glycerol, pimelic acid, suberic acid, azelaic acid, palmitic acid, and stearic acid) and the EA and MMA peaks of an EA/MMA acrylic binder. (**b**) Pyrogram of the white 0, orange 1, and violet 2 layers of the OS P5 sample acquired by TMH–GC/MS showing the most important marker peaks of a drying oil binder (glycerol, azelaic acid, palmitic acid, and stearic acid), the EA and MMA peaks of an EA/MMA acrylic binder, and the benzene, benzoic acid, methyl ester, pentaerythritol, dimethyl ester, azelaic acid, palmitic acid, and stearic acid peaks of an alkyd binder. Additionally, the marker peaks of rosin acids such as DHA (dehydroabietic acid), TDHA-7-M (tetradehydroabietic acid, 7-methoxy), and 7-oxo-DHA (7-oxodehydroabietic acid) are shown.

Regarding the organic compounds, Py–GC/MS analyses of OS P1 containing both the white ground and orange layers demonstrated the use of a drying oil and EA/MMA acrylic binder. Figure 13a shows the main EA and MMA monomer peaks of the acrylic binder, as well as the principal drying oil peaks detected in the OS P1 sample. The A/P (Azelaic/Palmitic) and P/S (Palmitic/Stearic) ratios were calculated in a similar manner to the method used for the monochrome canvas in order to determine the botanical origin of the drying oil. The A/P and P/S ratios corresponded respectively to 1.13 and 2.16 of the investigated OS P1 sample, which were in the range of the values for the specification of soya oil. Nevertheless, care should be taken in the interpretation of that result, since only one measurement was performed, thus not allowing for achieving reproducibility in the results. 

µ-ATR–FTIR analyses were performed on the cross-sectioned samples for determining whether the drying oil or acrylic binder belonged to the ground or orange layer. As shown in Figure 11, the ATR spectrum of white ground layer 0 matches the main IR bands of a drying oil binder, such as linseed oil (3008, 2921, 2851, 1740, 1718, 1455, 1399, 1245, and at 723 cm^−1^), thus demonstrating that the drying oil was part of the white ground layer 0. Furthermore, the IR band at 1547 cm^−1^ with a shoulder at 1527 cm^−1^ (asymmetric COO- stretching) complemented by two sharp peaks at 1455 and 1399 cm^−1^ (symmetric COO- stretching) (these latter two overlapping those of the drying oil) are characteristics of carboxylate products normally formed and found in oil paints. 

ORANGE LAYER: The orange layer applied to the white layer was composed of *cadmium sulfoselenide lithopone orange (CdS,xCdSe + BaSO_4_—Color Index PO20:1)* as the pigment and *EA/MMA acrylic* as the binder (copolymer of Ethyl Methacrylate and Methyl Methacrylate). 

**Figure 14:**
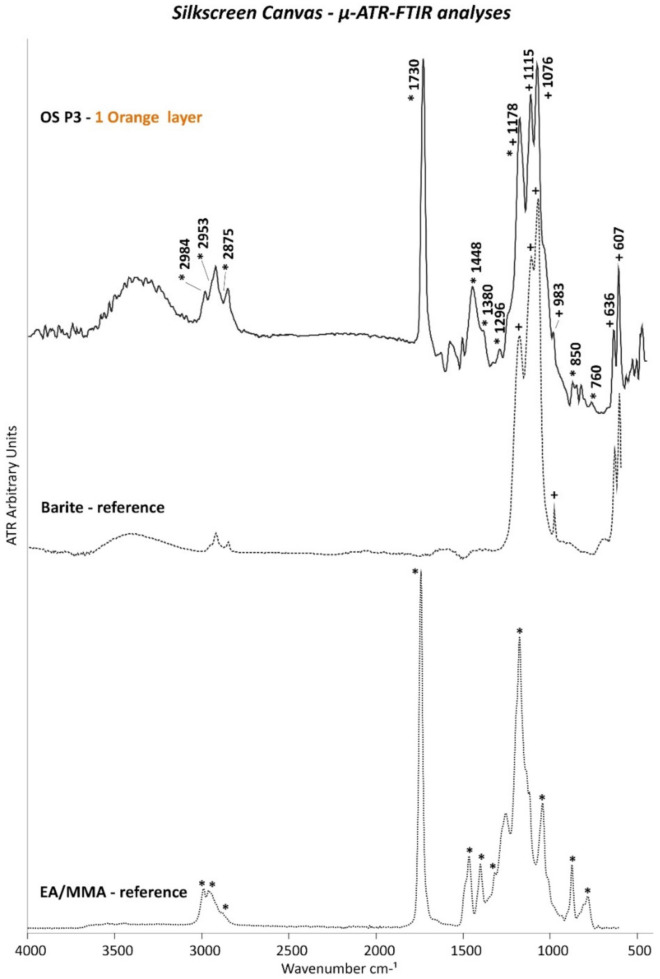
ATR spectra of orange layer 1 of OS P3. The ATR spectra of the EA/MMA (*) and barite (BaSO_4_) (+) references are also included.

The orange pigment type was identified by EDX analyses based on their main elements (Table 5), while µ-ATR–FTIR investigations confirmed the presence of barite (BaSO_4_) as part of the cadmium sulfoselenide lithopone orange pigment (Figure 14). The chemical composition of this orange pigment resulted to be similar to that of the first orange layer (2nd layer) from the monochrome canvas. 

Concerning the binder, the THM–GC/MS analyses carried out on OS P1 (white and orange layers) showed an EA/MMA type of acrylic binder in addition to a drying oil binder (Figure 13a). As discussed before, µ-ATR–FTIR analyses on the cross-sectioned samples permitted the localization of each organic binder to one of the two paint layers. Indeed, as shown in Figure 14 and similarly to the first orange layer of the monochrome canvas, the EA/MMA acrylic binder was found in the orange layer of the silkscreen canvas according to its characteristic IR bands (2984, 2953, 2875, 1730, 1448, 1380, 1296, 1178, 850, and 760 cm^−1^). 

**Figure 15:**
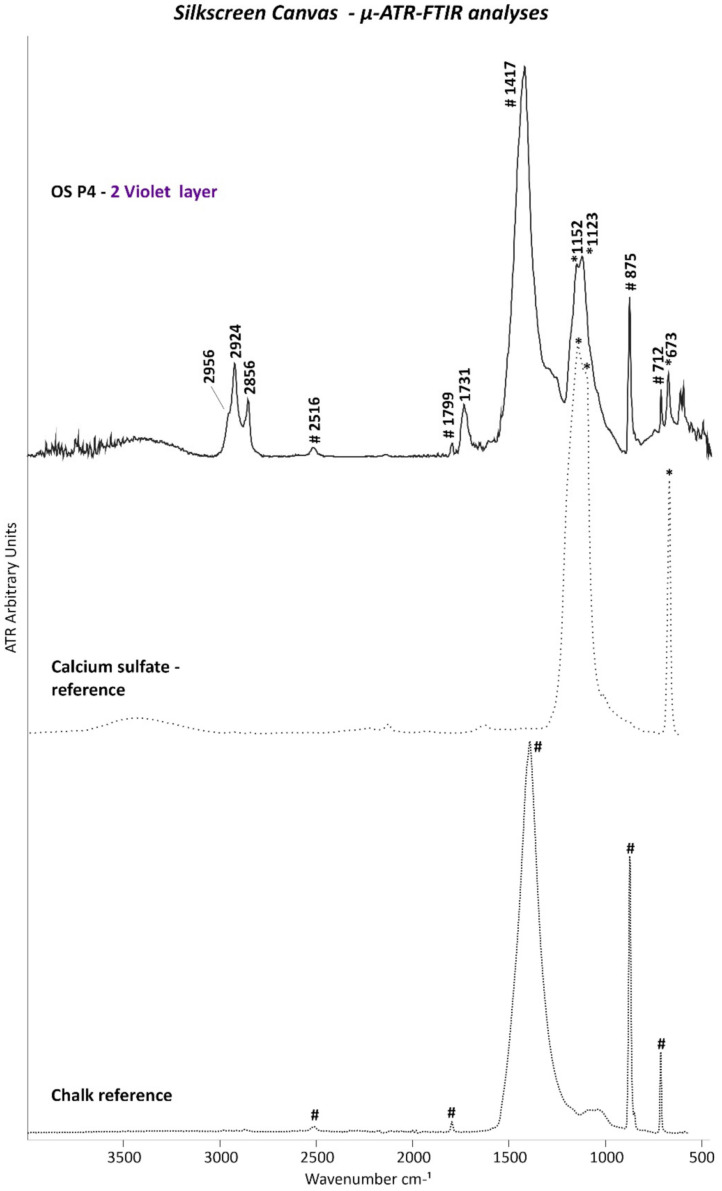
ATR spectra of violet layer 2 of OS P4. The ATR spectra of the chalk (CaCO_3_) (#) and calcium sulfate (CaSO_4_) (*) references are also included.

VIOLET LAYER: The violet layer of the OS P4 sample was constituted by a mixture of an *unidentified synthetic organic pigment or dye*, *chalk (CaCO_3_)* and *calcium sulfate (CaSO_4_)* as extenders, an *alkyd* as binder, and *rosin acids* as additives. EDX analyses registered the main elements of chalk (Ca) and calcium sulfate (Ca and S) (Table 5), while µ-ATR–FTIR and Raman measurements confirmed the presence of both extenders based on their main detected bands as shown in Figure 12 and Figure 15, respectively.

With respect to the organic components, an alkyd binder type and rosin acids were evidenced by THM–GC/MS analyses, as shown in Figure 12b, by the detection of their main characteristics pyrolysis peaks (benzoic acid-methyl ester with *m*/*z* = 51, 77, **105**, and 136; pentaerythritol-dimethyl ester with *m*/*z* = 55, 69, **75**, 85, 101, 114, and 128; phthalic acid, dimethyl ester with *m*/*z* = 77, 92, 135, **163**, 194; azelaic acid, palmitic acid, and stearic acid for alkyd; DHA (dehydroabietic acid) with *m*/*z* = 69, 117, 141, 173, 197, **239**, 299, and 314; TDHA-7-M (tetradehydroabietic acid, 7-methoxy) with *m*/*z* = 55, 126, 181, 209, 237, 267, 312, 327, and **342**; and 7-oxo-DHA (7-oxodehydroabietic acid) with *m*/*z* = 67, 115, 129, 187, 213, **253**, 269, 296, and 328 for rosin acids). The presence of rosin acids may be ascribable to their use as an additive for the violet color. Natural resins, such as rosin acids, from wood are usually used as portions of vehicles of printing inks [35]. Particularly, additives, such as colophonium resin (rosin), enhance the application properties, e.g., the dispersibility and color strength, of the pigment in the ink [36].

As reported in the white ground and orange layer discussion, the additional identification of EA/MMA and drying oil in OS P4 by THM–GC/MS is due to their presence in the orange and white ground layer, respectively, according to the µ-ATR–FTIR analyses.

Unfortunately, neither Py–GC/MS analyses nor Raman or µ-ATR–FTIR measurements could detect the type of material used for imparting the violet color. Based on the absence of main elements by EDX analyses and on the presence of rosin acids found by the THM–GC/MS investigations, it may be assumed that either a synthetic organic pigment or a dye represented the violet color. 

## 4. Conclusions

The different analytical techniques (OM under Vis and UV, SEM/EDX, Py–GC/MS, THM–GC/MS, µ-Raman, and µ-ATR–FTIR spectroscopies) employed for the analysis of the samples obtained from the monochrome and silkscreen canvases of “*Orange Car Crash*” by Andy Warhol provided fundamental information about their main inorganic and organic components. 

The obtained results indicated that the monochrome canvas was mostly characterized by two white ground layers: the one from the bottom was made of a mixture of zinc white (ZnO—Color Index PW4) as a pigment and linseed oil as a binder, while the upper ground layer (1st layer) was composed of titanium white (TiO_2_ rutile—Color Index PW6) and lithopone (barite (BaSO_4_) + zinc sulfide (ZnS)—Color Index PW5) as pigments and linseed oil as a binder as well. Above the second white ground layer, two different orange paint layers were identified: the first one was based on cadmium sulfoselenide lithopone orange (CdS,xCdSe + BaSO_4_—Color Index PO20:1) as a pigment and EA/MMA acrylic as a binder (copolymer of Ethyl Methacrylate and Methyl Methacrylate), and the second one (3rd layer) found in OM P6—likely related to a retouching—was also characterized by cadmium sulfoselenide lithopone orange (CdS,xCdSe + BaSO_4_—Color Index PO20:1) as a pigment and EA/MMA acrylic as a binder, but with the addition of chalk (CaCO_3_) as an extender. Furthermore, a coating/varnish of *n*BA/MMA acrylic (copolymer of *n*Butyl Methacrylate and Methyl Methacrylate) was identified. 

Regarding the silkscreen canvas, the used multi-analytical approach showed the application of only one white ground layer composed of titanium white (TiO_2_ rutile—Color Index PW6) and calcium sulfate (CaSO_4_) as an extender mixed with a soya oil binder. Only one orange layer was found made of cadmium sulfoselenide lithopone orange (CdS,xCdSe + BaSO_4_—Color Index PO20:1) as the pigment and EA/MMA acrylic as the binder, similar to the first orange layer of the monochrome canvas. The additional violet layer of the OS P4 sample was formed by an unidentified synthetic organic pigment or dye, chalk (CaCO_3_) and calcium sulfate (CaSO_4_) as extenders, an alkyd as the binder, and rosin acids as additives. 

The determined paint stratigraphy and chemical composition of each layer for each canvas clearly show the diversity in the materials, except for the orange paint layer. All the information obtained through these strategic, multi-analytical investigations have been decisive for devising conservation strategies about removing or reducing the detected *n*BA/MMA acrylic coating from the EA/MMA acrylic paint. At this stage, mock-ups are being fabricated mimicking the original materials and layer structure, on which tests of products and methods for removing or reducing the coating will be performed in the next step. 

Additionally, this work provides valuable and rare scientific information about one of Andy Warhol´s artwork. The results here gained may be of help for possible future research studies on further famous masterpieces of Andy Warhol.

## Figures and Tables

**Table 1:** List of the samples taken from the monochrome and silkscreen canvases of “*Orange Car Crash*” by Andy Warhol, including the names, description, sampling area, preparation type, and the employed analytical techniques used for their investigation. ✓ = performed; 🗴 = not performed.

Canvas	Sample Name	Description	Sampling Area	Sample Preparation	OM	SEM/EDX	Py-GC/MS	THM-GC/MS	µ-ATR-FTIR	Raman
**Monochrome**	OM P1	Orange paint and ground layer	2 samples from the top foldover edge on the reverse, 152 and 154 cm from the left	🗴	🗴	🗴	✓	🗴	🗴	✓
	OM P2	Orange paint layer	Loose paint flake on the left tacking margin, 128 cm from the top	🗴	🗴	🗴	✓	🗴	🗴	✓
	OM P3	White ground layer	From the left tacking margin, 145.5 cm from the top	🗴	🗴	🗴	🗴	✓	🗴	✓
	OM P4	Coating (presumably from restoration 1993)	From various areas in the bottom half of the painting, close to the left edge	🗴	🗴	🗴	✓	🗴	🗴	🗴
	OM P5	Orange paint and ground layer	From a loss on the tacking margin on the right side, 144 cm from the top	Cross-section	✓	✓	🗴	🗴	✓	✓
	OM P6	Orange paint and ground layer	From the tacking margin on the right side, 24.9 cm from the top	Cross-section	✓	✓	🗴	🗴	✓	✓
**Silkscreen**	OS P1	Orange paint and ground layer	From the bottom foldover edge on the reverse: 73.3 cm from the left and 2 cm from the bottom	🗴	🗴	🗴	✓	🗴	🗴	✓
	OS P2	Violet silkscreen paint	24.8 cm from the left and 120.3 cm from the bottom	🗴	🗴	🗴	✓	✓	🗴	✓
	OS P3	Orange paint and ground layer	115.9 cm from the bottom and 1.4 cm from the right	Cross-section	✓	✓	🗴	🗴	✓	✓
	OS P4	Violet silkscreen paint, orange paint and ground layer	First silkscreen image bottom left: 96 cm from the bottom and 0.2 cm from the left	Cross-section	✓	✓	✓	✓	✓	✓
	OS P5	Orange paint and ground layer	From the foldover edge on the reverse: 73.3 cm from the left and 2 cm from the bottom (same area as for OS P1)	Cross-section	✓	✓	🗴	🗴	✓	✓

**Table 2:** ID of the main components identified for each layer of the monochrome canvas by different analytical techniques.

Canvas	Paint Layer	Color	Pigment Color Index	ID by SEM/EDX	ID by Py-GC/MS	ID by THM-GC/MS	ID by µ-ATR-FTIR	ID by Raman
**Monochrome**	0	White	PW4	Zinc white (ZnO)	🗴	Linseed oil	Linseed oil	🗴
	1	White	PW6–PW5	Titanium white (TiO_2_), lithopone (barite (BaSO_4_) + zinc sulphide (ZnS)	🗴	Linseed oil	Linseed oil traces, barite (BaSO_4_) as part of lithopone	Titanium white (TiO_2_) (rutile), lithopone (barite (BaSO_4_) + zinc sulphide (ZnS)
	2	Orange (retouch)	PO20:1	Cadmium sulfoselenide lithopone orange (CdS,xCdSe + BaSO_4_)	EA/MMA acrylic	🗴	EA/MMA acrylic-barite (BaSO_4_) as part of cadmium sulfoselenide lithopone	🗴
	3	Restoration overlay	PO20:1–PW18	Cadmium sulfoselenide lithopone Orange (CdS,xCdSe + BaSO_4_), chalk (CaCO_3_)	EA/MMA acrylic	🗴	EA/MMA acrylic-barite (BaSO_4_) as part of cadmium sulfoselenide lithopone,	🗴
	4	Transparent (coating)	🗴	🗴	nBA/MMA acrylic	🗴	🗴	🗴

**Table 3:** Elements detected by EDX per layer on the cross-sectioned OM P5 and OM P6 samples. Main elements are written in bold. (+++ high, ++ moderate, + low concentration).

Canvas	OM P5—Layer	Elements
**Monochrome**	0—White	**Zn (+++)**
	1—White	**S (+++), Ti (++), Zn (++), Ba (+),** Cd (trace)
	2—Orange	**S (+++), Cd (++), Ba (++), Se (trace),** Zn (+)
	**OM P6—Layer**	**Elements**
	0—White	**Zn (+++),** Ti (trace)
	1—White	**S (+++), Ti (+++), Zn (++), Ba (+)**
	2—Orange	**S (+++), Cd (++), Ba (++), Se (+),** Ti (trace), Zn (trace)
	3 (a & b)—Orange	**S (+++), Cd (++), Ba (++), Ca (++), Se (+),** Zn (trace)

**Table 4:** ID of the main components identified for each layer of the silkscreen canvas by Andy Warhol by different analytical techniques.

Canvas	Paint Layer	Color	Pigment Color Index	ID by SEM/EDX	ID by Py-GC/MS	ID by THM-GC/MS	ID by µ-ATR-FTIR	ID by Raman
**Silkscreen**	0	White	PW6–PW25	Titanium white (TiO_2_), calcium sulphate (CaSO_4_)	🗴	Soya oil	Drying oil, calcium sulphate (CaSO_4_)	Titanium white (rutile) (TiO_2_), calcium sulphate (CaSO_4_)
	1	Orange	PO20:1	Cadmium sulfoselenide lithopone orange (CdS,xCdSe + BaSO_4_)	EA/MMA acrylic	EA/MMA acrylic	EA/MMA acrylic, barite (BaSO_4_) as part of lithopone	🗴
	2	Violet	Unidentified	Calcium sulphate (CaSO_4_), chalk (CaCO_3_)	🗴	Alkyd, rosin acids	Calcium sulphate (CaSO_4_), chalk (CaCO_3_)	Calcium sulphate (CaSO_4_), chalk (CaCO_3_)

**Table 5:** Elements detected by EDX per layer on the cross-sectioned OS P3, OS P4, and OS P5 samples. The main elements are written in bold. (+++ high, ++ moderate, + low concentration, tr. trace).

Canvas	OS P3—Layer	Elements
**Silkscreen**	0—White	**S (+++), Ca (++), Ti (++),** Si (tr.), As (tr.)
	1—Orange	**S (+++), Cd (++), Ba (++),** Ca (+), **Se (tr.),** Si (tr.)
	**OS P4—Layer**	**Elements**
	0—White	**S (+++), Ca (++), Ti (++),** Si (tr.), As (tr.)
	1—White	**S (+++), Cd (++), Ba (++),** Ca (+), Ti (+), **Se (tr.),** Si (tr.),
	2—Violet	**Ca (+++), S (+),** Ti (+), Si (tr.)
	**OS P5—Layer**	**Elements**
	0—White	**S (+++), Ca (++), Ti (++),** Si (tr.), As (tr.)
	1—Orange	**S (+++), Cd (++), Ba (++),** Ca (++), **Se (tr.),** Ti (tr.), Si (tr.)

## Data Availability

The most significant data generated or analysed during this study are included in this published article. Further results obtained during the current study are available from the corresponding author on reasonable request.
